# On the Present State of Homœopathy in Germany

**Published:** 1847-03

**Authors:** A. Mühry

**Affiliations:** Hanover


*On the present state of Homœopathy in Germany.* By Dr. A. Mühry, of Hanover.—Homœopathy has undoubtedly taught us, that however high an estimate medical men may have before formed of the healing powers of Nature, these must be rated much higher still. This fact may indeed render us more modest, but need not depress us into spiritless unbelievers. If, on the one hand, we ought to feel ashamed that the curative powers of Nature, aided—by nothing at all—should sometimes achieve as much as we are able to do ourselves, the history of homœopathy has, on the other hand, shown this system to be inadequate to the wants of human society; and has brought into stronger light, not merely the nullities of medicine, but also as a secondary result, its positive utility.

I think I am right in affirming that the history of homœopathy can be better traced in Germany, where the system originated (fifty years ago), and where its development took place, than in England, where it has not been practised more than from ten to fifteen years. It appears to me, likewise, that the changes wrought in homœopathy have not been sufficiently taken into account in your essay; and hence it may here be of some use to point out some of the principal epochs of the system.

Samuel Hahnemann published his 'Organon' of Medicine in the year 1810, but had begun to make his system known before that time. I think with you that he was a sincere believer in the truth of his doctrine. It is but just, however, to remark, that, at least once previously, he had deceived the world, by selling at a high price, under the name of *pnoeum*, a nostrum which consisted of nothing but *borax*. This is a fact undenied even by his adherents. He had before this published a pharmacological dictionary, and his system was altogether the offspring rather of Pharmacy than of Medicine, properly so called.

Among others, the following retractions and retrogressions have been made in the original doctrines of homœopathy, by various adherents. At first it was deemed requisite to administer medicines only once a week or once a fortnight. At present scarcely a single homœopathist adheres to this; but, on the contrary, now frequently orders them several times daily. A contradiction was also recognized in the assumption of a force-creating power of remedies by attenuation—a greater efficacy in less attenuated remedies. At the present day there is often administered a drop of unattenuated and unpotentialized tincture (termed elementary, or mother tincture.) Originally, the abstraction of blood, emetics, aperients, mineral waters and baths, were all proscribed: now they are often acknowledged as useful. Hahnemann's later doctrines, as promulgated in his 'Chronic Diseases,' met with few adherents: he had become inconsistent, and became more so still when he recommended camphor in very large, unattenuated doses against cholera. His successors became more and more divided, modifying the system in various ways, until its original meaning was almost entirely lost. This became apparent at a congress of homœopathic physicians, which took place at Magdeburg, either in 1836 or 1838. Their confession of faith attests how much reform and reaction the doctrine of numerous homœopathists had suffered. A fraction of the school has even laid aside the name of homœopathy, substituting for it that of "Specific Medicine." This sect is numerous in Germany, in the Grand Duchy of Baden. These still hold fast to the principle, "similia similibus," as explanatory of the curative process, but they no longer give the remedies after the original method. They busy themselves in experimenting with medicines upon healthy persons, but in large doses, and from this they look forward to grand results, which are, however, not perceptible as yet. They likewise attend to the physiological relations of the diseased body, and they have among their body some most able and respectable men. For at least ten years past, this "Specific Medicine" has supported a journal.

We may regard the homœopathists as sincere; but it is true that their number has augmented less by adoption of the original doctrine than of its modifications. They bear the original name wrongly, being no longer true homœopathists. The majority have even resumed a great portion of the allopathic medicines.

I do not hesitate to say that, although men of worth are to be found among them, there prevails, generally speaking, both within and without the profession, a low opinion of the standard of their intelligence. In Germany, no man of undoubted eminence has ever become a convert to the system. Only once has an instance like that of Professor Henderson occurred: Dr. Kopp, of Hanau, unexpectedly published a volume of "Practical Observations," descriptive of a series of homœopathic cases and cures of his own witnessing. He thus stamped himself a homœopathist. A few years afterward, another volume of the "Observations" appeared, communicating a further series of cases and cures—but of an allopathic nature. The author thus declared himself reconverted, and he has remained so ever since.

With us in Germany there are in almost every largish town one or more homœopathists, who are perhaps consulted in chronic cases, after various methods had been previously tried, and where it is thought that homœopathy, at any rate can do no *positive* harm. True it is, that cures are sometimes thus effected, and these create a sensation; while the cures accomplished by ordinary medicine pass unnoticed. A large proportion of homœopathists have recently adopted the hydropathic method of treatment. I am not cognizant of any town where homœopathy is exclusively practised.

If homœopathy has taught us that our curative control over the course of disease is far below what we rated it at, it has, *à fortiori*, though unintentionally, taught us that our "apparatus medicaminum" possesses less merit than we imagined, and that it need no longer continue as gross and rude as the instrument of an "armentarium chirurgicum" of old. It has, however, at the same time enabled us to mark with greater distinctness those diseases in which our remedies really effect the cure. Such are, for example, intermittent fevers, scabies, syphilis. These it was that first showed the insufficiency of homœopathy; and you will find that in the statistic bulletin of Dr. Fleischmann these diseases are omitted.

One thing certain is, that through means of homœopathy, medicine has undergone, is undergoing, and—as in your treatise you predict—will, in the future, yet further undergo a rigorous scrutiny. It appears to me, however, that rational, scientific medicine has, in some degree, already passed this ordeal; and if it can never claim the exactness of chemistry and mechanics, it may yet hope, in point of accuracy, to reach the standard attained by modern physiology.—*British and Foreign Medical Review.*

June 22, 1846.

